# Epidermolysis Bullosa With Congenital Absence of Skin: Congenital Corneal Cloudiness and Esophagogastric Obstruction Including Extended Genotypic Spectrum of *PLEC*, *LAMC2*, *ITGB4* and *COL7A1*

**DOI:** 10.3389/fgene.2022.847150

**Published:** 2022-04-01

**Authors:** Pharuhad Pongmee, Sanchawan Wittayakornrerk, Ramrada Lekwuttikarn, Sasikarn Pakdeeto, Piangor Watcharakuldilok, Chatchay Prempunpong, Thipwimol Tim-Aroon, Chawintee Puttanapitak, Piyawan Wattanasoontornsakul, Thitiporn Junhasavasdikul, Parith Wongkittichote, Saisuda Noojarern, Duangrurdee Wattanasirichaigoon

**Affiliations:** ^1^ Department of Pediatrics, Faculty of Medicine Ramathibodi Hospital, Mahidol University, Bangkok, Thailand; ^2^ Chakri Naruebodindra Medical Institute, Faculty of Medicine Ramathibodi Hospital, Mahidol University, Samut Prakan, Thailand; ^3^ Department of Pediatrics, Buriram Hospital, Buriram, Thailand; ^4^ Department of Surgery, Faculty of Medicine Ramathibodi Hospital, Mahidol University, Bangkok, Thailand; ^5^ Department of Pediatrics, Maharat Nakhon Ratchasima Hospital, Nakhon Ratchasima, Thailand; ^6^ Department of Radiology, Faculty of Medicine Ramathibodi Hospital, Mahidol University, Bangkok, Thailand; ^7^ Division of Genetics and Genomic Medicine, Department of Pediatrics, Washington University School of Medicine, St. Louis, MO, United States

**Keywords:** bilateral hydronephrosis, cloudy cornea, epidermolysis bullosa with pyloric atresia, intestinal obstruction, reflux nephropathy

## Abstract

Epidermolysis bullosa (EB) is a rare and genetically heterogeneous disorder characterized by skin fragility and blister formation occurring spontaneously or after minor trauma. EB is accompanied by congenital absence of skin (EB with CAS) in some patients. Pathogenic variants of *COL7A1* are responsible for EB with CAS in the vast majority of cases. Type and subtype diagnosis of EB with CAS generally requires specific immunohistological examinations that are not widely available plus targeted gene analysis. The present study aimed to determine the clinical features of five patients affected by EB with CAS and to identify the underlying genetic defects using whole exome sequencing (WES) followed by focused analysis of the target genes. Four patients had generalized skin involvement and one had localized defects. Two patients exhibited extremely severe skin manifestations and congenital cloudy cornea along with pyloric atresia, and one had partial esophagogastric obstruction and anuria due to vesicoureteric obstruction. In the WES analysis, the average coverage of the target exons was 99.05% (726 of 733 exons), with a range of 96.4–100% for individual genes. We identified four novel and two known pathogenic/likely pathogenic variants of five distinct genes in the examined families: *PLEC*:c.2536G > T (p.Glu846Ter); *LAMC2*:c.3385C > T (p.Arg1129Ter); *KRT5*:c.429G > A (p.Glu477Lys); *ITGB4:*c.794dupC (p.Ala266SerfsTer5); *COL7A1*:c.5440C > T (p.Arg1814Cys); and *COL7A1*:c.6103delG. All alleles were inherited from the parents, except for the *KRT5* variant as a *de novo* finding. The findings reveal extremely rare phenotypes found in EB with CAS, namely congenital cloudy cornea, esophagogastric obstruction, and anuria, and extend the genotypic spectrum of EB-related genes. The data confirm that WES provides very high coverage of coding exons/genes and support its use as a reasonable alternative method for diagnosis of EB. The present data from an underrepresented population in Southeast Asia could further broaden the knowledge and research on EB.

## Introduction

Epidermolysis bullosa (EB) is an inherited mechanobullous disorder characterized by skin fragility and blister formation occurring spontaneously or after minor trauma. It is classified into four main types based on the level of separation of the skin: EB simplex (EBS), junctional EB (JEB), dystrophic EB (DEB), and Kindler EB (KEB) (Has et al., 2020a). EB is known to exhibit high genetic heterogeneity.

EBS is the most common form of EB and is characterized by blister formation within the epidermis arising from cleavage within the basal layer. Most cases of EBS are caused by dominant mutations of *KRT5* (keratin 5), *KRT14* (keratin 14), and *KLHL24*, with a few cases linked to recessive variants of *PLEC*, *KRT5*, *KRT14*, and other genes (Fine et al., 2014; Has et al., 2020b; Mariath et al., 2020). JEB involves separation between the dermis and the epidermis (lamina lucida) and is mostly inherited in an autosomal recessive mode. DEB is characterized by separation within the uppermost dermis (sublamina densa) and has two subtypes, autosomal dominant DEB (DDEB) and autosomal recessive DEB (RDEB), that both arise from mutations in *COL7A1* (collagen type VII). KEB affects mixed layers of the skin and is associated with photosensitivity (Has et al., 2020a).

EB with congenital absence of skin (CAS), previously named Bart syndrome, is considered a clinical finding under the umbrella of EB (Fine et al., 2014; Has et al., 2020b). EB with CAS can be diagnosed based on clinical findings. However, identification of the layer of skin cleavage and precise classification of the EB type/subtype require immunofluorescence mapping and/or transmission electron microscopy, preferably on newly-induced blisters (Has et al., 2020a).

The most common EB type associated with CAS is DEB (58–64%) arising from *COL7A1* mutations, followed by JEB (linked to *ITGB4*) and EBS in that order (Mariath et al., 2020; Martinez-Moreno et al., 2020). Recessive variants of *LAMC2* (laminin-332), *ITGA6* and *PLEC* are less frequently found in these cases (Mariath et al., 2020; Martinez-Moreno et al., 2020). Autosomal dominant EB with CAS is mainly caused by monoallelic variants of *KRT5* and *KLH24*. EBS-severe with pyloric atresia (EBS-PA) is linked to mutations in *PLEC*, while JEB-PA is caused by mutations in *ITGB4* and rarely *ITGA6* (Pfendner and Uitto, 2005; Natsuga et al., 2010a; Natsuga, 2015; Mariath et al., 2020).

Herein, we describe EB with CAS in five unrelated patients, revealing rare phenotypes of congenital cloudy cornea, esophagogastric obstruction, and anuria and the underlying genetic defects.

## Methods

### Patients

We encountered five patients with a clinical diagnosis of EB with CAS during 2014–2020. Clinical and laboratory data were collected from their medical records. All of the patients and their parents were of Thai descent.

Peripheral blood samples from the index cases and their parents were obtained for genetic analysis, after written informed consent was received. The research protocol was approved by the Ramathibodi Hospital Human Research Ethics Committee (approval number: MURA 2020/837)*.* Individual written consent for photograph and publication was obtained.

### Whole Exome Sequencing and Data Analysis

DNA was extracted from the peripheral blood samples using a Gentra® Puregene® kit (QIAGEN®, Hilden, Germany). WES was performed on an IIumina HiSeq4000 or NovaSeq platform by Macrogen® (Seoul, Republic of Korea) using Agilent SureSelect (V5+UTR) for target captures (∼22,000 genes; 100-bp pair-end mode and 125x coverage of target regions). Analysis of the raw genome sequencing data was as described in the Supplementary material and previous established methods (Van der Auwera et al., 2013).

Twenty-six genes known to be associated with EB were analyzed: *ATP2C1*, *CDSN*, *COL17A1*, *COL7A1*, *CSTA*, *DSG1*, *DSG2*, *DSG4*, *DSP*, *DST*, *EXPH5*, *FERMT1*, *GRIP1*, *ITGA3*, *ITGA6*, *ITGB4*, *KLHL24*, *KRT1*, *KRT5*, *KRT14*, *LAMA3*, *LAMB3*, *LAMC2*, *PKP1*, *PLEC*, and *TGM5*. These genes were chosen following one of the widely available commercial panels for epidermolysis bullosa (Blueprint Genetics®, https://blueprintgenetics.com).

Variants with minor allele frequency (MAF) > 0.05 were filtered out, in accordance with the population database (http://gnomad.broadinstitute.org/) and 2015 guidelines of the American College of Medical Genetics and Genomics and the Association of Molecular Pathology (ACMG/AMP) (Richards et al., 2015). Subsequently, the frequencies of the identified variants were checked against the Thai Reference Exome Database (T-REx, https://trex.nbt.or.th/).

The pathogenicities of the identified variants were determined using previous reports and disease databases including ClinVar (https://www.ncbi.nlm.nih.gov/clinvar/), the Human Gene Mutation Database (HGMD), and the ACMG/AMP guidelines (Richards et al., 2015). The searches were performed using a variant prediction software, VarSome, (https://varsome.com/), that incorporates a set of 14 computational prediction programs (REVEL, DANN, DEOGEN2, FATHMM-MKL, LIST-S2, M-CAP, MVP, PolyPhen2, MutationTaster, BayesDel_addAF, EIGEN, MutationAssessor, PrimateAI and SIFT) for prediction of pathogenicity.

Sanger sequencing was carried out to confirm the alleles identified by WES in the patients and their family members. Segregation analysis was performed to determine the inheritance pattern. Primers with intronic flanking sequence were designed using PRIMER3 software (http://frodo.wi.mit.edu). Lists of the primer sequences and GenBank reference sequences are provided in the Supplementary Table S1.

## Results

### Clinical Data

**FIGURE 1:**
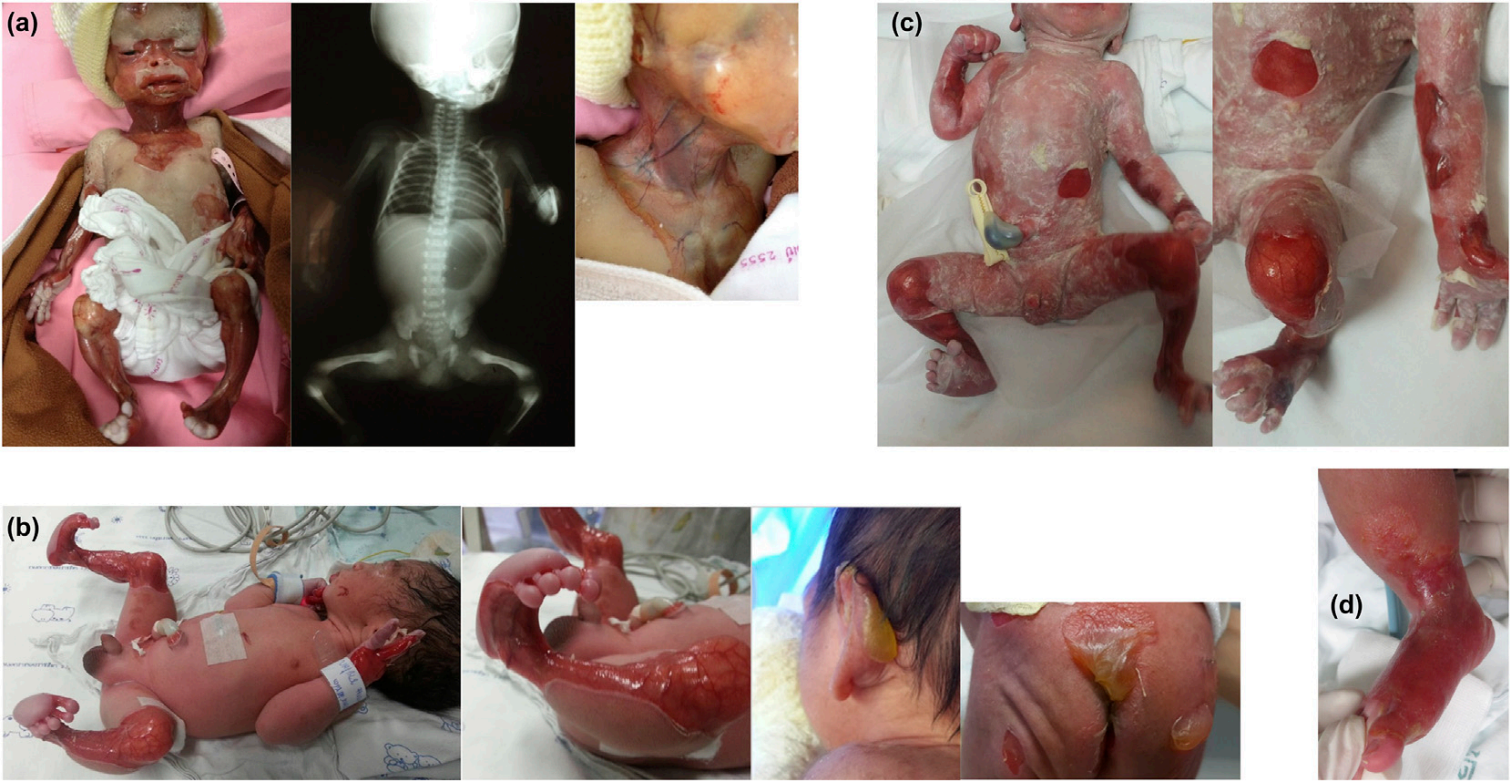
Characteristics of skin involvement and other abnormalities of Patients 1–3 and 5. **(A)** Patient 1: marked multiple absence of skin and well-demarcated erythematous atrophic patches on the face, neck, upper chest wall, and upper and lower extremities; and x-ray showing single large gastric bubble suggesting pyloric atresia. **(B)** Patient 2: well-demarcated erythematous atrophic patches and absence of skin on both legs, extending to the feet, and tense large bullae at the posterior aspect of left ear pinna, sacral area, and buttocks. **(C)** Patient 3: notable well-defined erythematous atrophic patches and absence of skin at the abdominal wall, forearms, and both legs. **(D)** Patient 5: localized absence of skin at the dorsum of the right foot up to the ankle.

**FIGURE 2:**
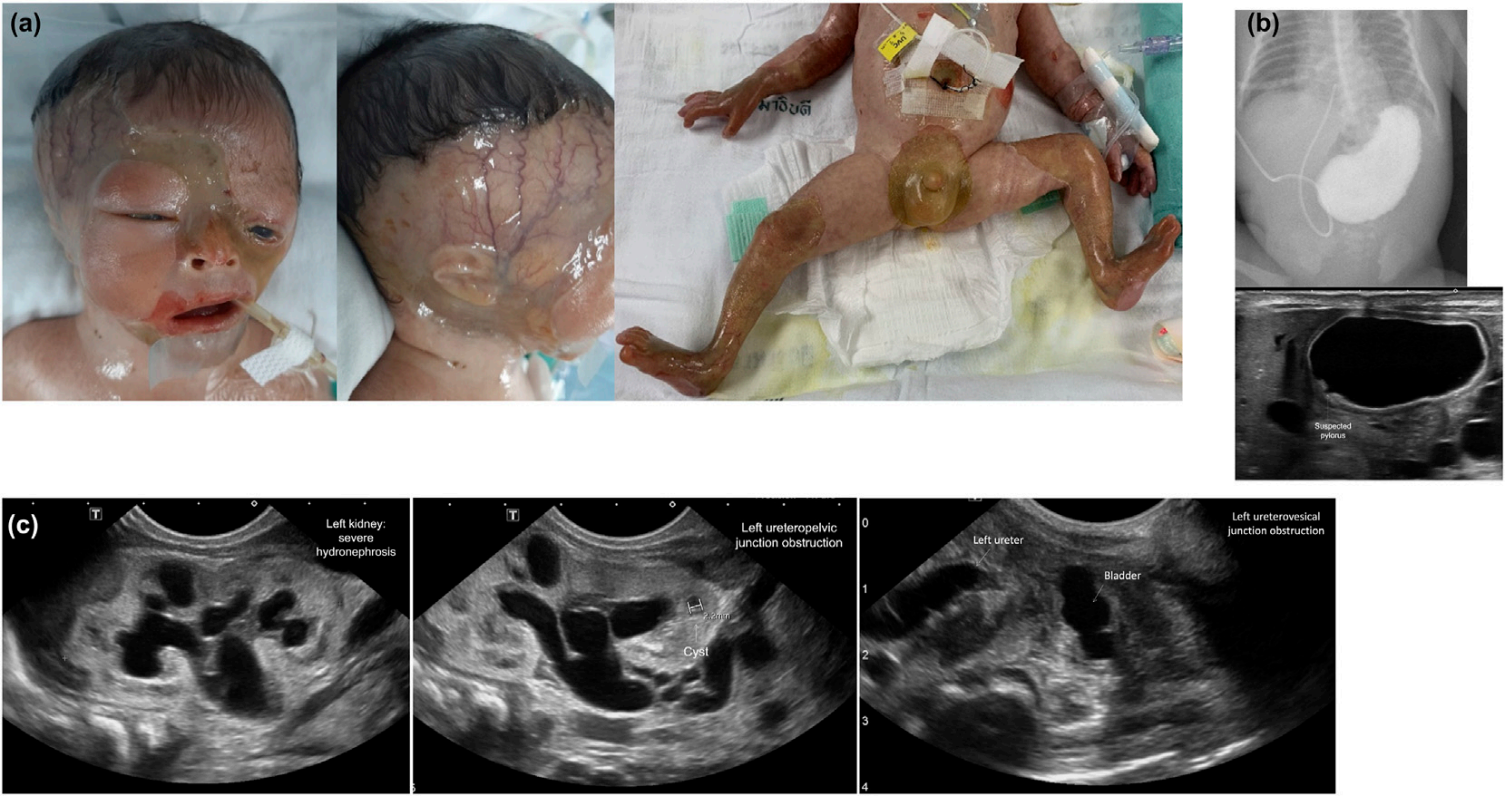
Characteristics of skin involvement and other abnormalities of Patient 4. **(A)** Skin involvement: marked complete absence of skin over the parietotemporal region of the scalp and extending downward to the interorbital and midfacial areas including the nasal region; perineal area; upper and lower extremities including thighs, legs, feet, arms, forearms, and hands; bilateral cloudy cornea, ectropion of the left lower eyelid and dysplastic ears. **(B)** Pyloric atresia: an upper gastrointestinal study showing a gastric outlet obstruction and abdominal ultrasound showing large air-filled stomach. **(C)** Abdominal ultrasound demonstrating multi-leveled urinary tract obstructions: severe hydronephrosis of the left kidney (far left); left ureteropelvic junction obstruction (middle); and a small and collapsed urinary bladder and markedly dilated left ureter, signifying a vesicoureteric junction obstruction (far right). Similar findings were observed on the right kidney and ureter (data not shown).

Five patients (4 males, one female) were enrolled in the study. None of the patients had histological confirmation of the EB type diagnosis because a specific immunohistopathological diagnostic facility was unavailable. The characteristics of the five patients and pedigrees and their skin lesions are described below and/or shown in Table 1 and Figures 1, 2, and the Supplementary Figure S1.

**TABLE 1:** Patients’ clinical characteristics including outcomes and responsible genetic defects.

Characteristics	Patient 1	Patient 2	Patient 3	Patient 4	Patient 5
Gestational age (wk)	34	35	33	31	39
Birth weight (g)	2,000	1,800	1,627	1,650	3,335
Sex	Female	Male	Male	Male	Male
Parental consanguinity	Yes	No	No	No	No
Skin/oral findings					
Aplasia cutis: distribution	Generalized: extensive over face, neck, chest, UE, LE	Generalized: extensive over UE, LE	Generalized: extensive over abdomen, UE, LE	Generalized: extensive: over scalp, face, neck, UE, LE, perineum	Localized: right ankle
Blister formation: Onset	DOL1 (few hr)	DOL4	DOL1	DOL2 (36 h)	DOL 1
Oral blister	No?	Yes	Yes	No	Yes
Other areas	No	Hands, elbow, buttocks, post-auricular; later over the entire body	Abdominal wall, upper thighs, feet, later over the entire body	Periumbilical, areas attached with adhesive	Extremities
Atrophic scar	NA	NA	Yes	NA	No
Dystrophic nails	NA	No	No	Yes	No
Reticulated erythema	NA	Yes	Yes	NA	No
Extracutaneous features					
Eye	Congenital cloudy cornea	No	No	Congenital cloudy cornea, absent eyelashes	No
Gastrointestinal	Pyloric atresia	Difficulty sucking	Difficulty sucking	Partial esophagogastric obstruction, pyloric atresia	No
Urogenital	No	No	No	Urethral meatal stricture, vesicoureteral stricture, hydroureter, anuria	No
Musculoskeletal	Ankle contracture	Ankle contracture	No	No	No
Outcomes: age, clinical course	Dead: DOL1, breathing stopped	Dead: DOL35, sepsis (catheter-related)	Dead: DOL44, sepsis, palliative care	Dead: DOL2, acute kidney injury, anuria	Alive:11 mo, discharged on DOL10
Genetic defects	*PLEC*	*LAMC2*	*KRT5*	*ITGB4*	*COL7A1*

DOL, day of life; hr, hours; LE, lower extremities; mo, months; NA, not available; UE, upper extremities; wk, weeks.

### Patient 1

Patient 1 was a 34-weeks gestational age (GA) female neonate born to a G4P3 woman. Her birth weight was 2,000 g and she had Apgar scores of 9 and 10 at 1 and 5 min, respectively. Physical examination revealed hypotonia and extensive CAS with well-demarcated erythematous atrophic patches on her neck, trunk, and upper and lower extremities, including the hands, legs and feet. There was large erosion at the central part of the face, infraorbital area, and nose. Cloudy cornea was noted. A babygram was suggestive of pyloric atresia. The patient developed multiple bullae at different sites of the entire body, and died a few hours after birth. The parents were third cousins and were 42 years of age. Their first and third children were healthy, while the second child was affected by extensive skin defects at birth and died on the first day of life (DOL), with no available medical records or photographs for review.

### Patient 2

Patient 2 was a male infant, first child of the family, born at 35 weeks GA with a birth weight of 1,800 g and Apgar scores of 9 and 10 at 1 and 5 min, respectively. Physical examination showed generalized well-demarcated absence of skin over the dorsum of the hands, the anteromedial aspect of the legs and the dorsum of the feet. Both ankles were in a fixed dorsiflexion position. No nail deformity was noted. On DOL4, multiple bullae formed on his fingers, elbows, buttocks, and left postauricular area, and subsequently developed over the whole body. General supportive treatment and antibiotics were given. However, at 1 month of age, the patient exhibited high fever and had pus oozing from the umbilical venous catheter insertion site, resulting in sepsis and death. The parents were aged 17 and 23 years at the time of his birth and had no known consanguinity despite originating from a close district.

### Patient 3

Patient 3 was a 33 weeks GA male neonate who was vaginally delivered by a G1P0 woman aged 32 years. The pregnancy was complicated by gestational diabetes and preterm labor pain and membrane rupture with clear amniotic fluid for 2 days before delivery, necessitating intrapartum antibiotic prophylaxis and antenatal steroid to promote fetal lung maturity. The Apgar scores were eight and nine at 1 and 5 min, respectively, and the birth weight was 1,627 g. The infant had multiple well-defined erythematous atrophic patches and absence of skin noted at the abdominal wall, arms, and legs. At 12 h after birth, he developed multiple thin bullae at the abdominal wall, upper thighs, and pressured areas, with subsequent peeling off of the skin. Subsequent development of intraoral blisters led to erosive lesions and sucking difficulty, necessitating orogastric tube feeding. Despite provision of extensive wound care, progressive scaring and recurrent bacterial sepsis followed, leading to the decision for palliative care which resulted in subsequent death on DOL44. Both parents were 32 years of age and were a nonconsanguineous couple.

### Patient 4

Patient 4 was noted to have fetal bilateral hydronephrosis and hydroureter, as demonstrated by routine prenatal ultrasound at 29 weeks. This male neonate was born at 31 weeks GA by cesarean section due to preterm labor pain and premature membrane rupture. He had Apgar scores of 4 and 10 at 1 and 5 min, respectively, and his birth weight was 1,650 g. There was widespread complete absence of skin over the parietotemporal region of the scalp, interorbital and midfacial area including the nasal region, perineal area, and upper and lower extremities including the thighs, legs, feet, arms, forearms, and hands. Bilateral cloudy cornea, ectropion of the left lower eyelid, and dysplastic ears were noted. There were dystrophic nails and loss of the nail plate on the second and third fingers of the left hands. Bilateral palpable flank masses, absent urethral meatus, anuria, and elevated serum creatinine level indicating acute kidney injury were also noted. Abdominal ultrasonography revealed small-sized bladder, severe hydroureter and hydronephrosis, suggesting obstruction at the vesicoureteric junction. There were difficulty passing an orogastric tube gauge #6 farther than 15 cm from the oral opening, raising suspicion of a stricture at the esophagogastric junction. An orogastric tube gauge #10 was successfully passed to the stomach. A plain X-ray of the abdomen, ultrasonography, and upper gastrointestinal study were performed and demonstrated a pyloric stricture. On DOL2, the patient developed multiple peeled-off skin areas and a few blisters, particularly around the application sites of adhesive materials. The patient expired on the same day. The was no parental consanguinity.

### Patient 5

Patient 5 was a 39 weeks GA male infant who was vaginally delivered by a G1P0 woman aged 24 years. The Apgar scores were 9 and 10 at 1 and 5 min, respectively, and the birth weight was 3,355 g. At birth, he was noted to have localized CAS at the dorsum of the right foot up to the ankle, with two large blebs on his upper and lower gums. The patient was discharged to home on DOL7. The CAS lesion gradually improved with complete healing by 3 weeks. Skin blistering did not appear until 3 months of age, when he developed small blebs on his right ankle and his gums, requiring local skin care that led to healing without scars. No milia formation or skin reticulation was observed. At the time of this report, the patient is 10 months of age with normal growth and development. His family history indicates no parental consanguinity. His father was 28 years of age.

### Whole Exome Sequencing Data and *PLEC*, *LAMC2*, *KRT5*, *ITGB4* and *COL7A1* Variants

Given the lack of available specimens for Patient 1, we used blood samples from his parents for a family duo WES analysis and two unaffected siblings for additional segregation analysis. For Patients 2–5, blood samples from the patients only underwent singleton WES to reduce costs.

The average coverage of the target exons by WES was 99.05% (726 of 733 exons), with a range of 96.4–100% for individual genes. The coverage and missing regions for the target genes are provided in the supporting information Supplementary Table S2.

**FIGURE 3:**
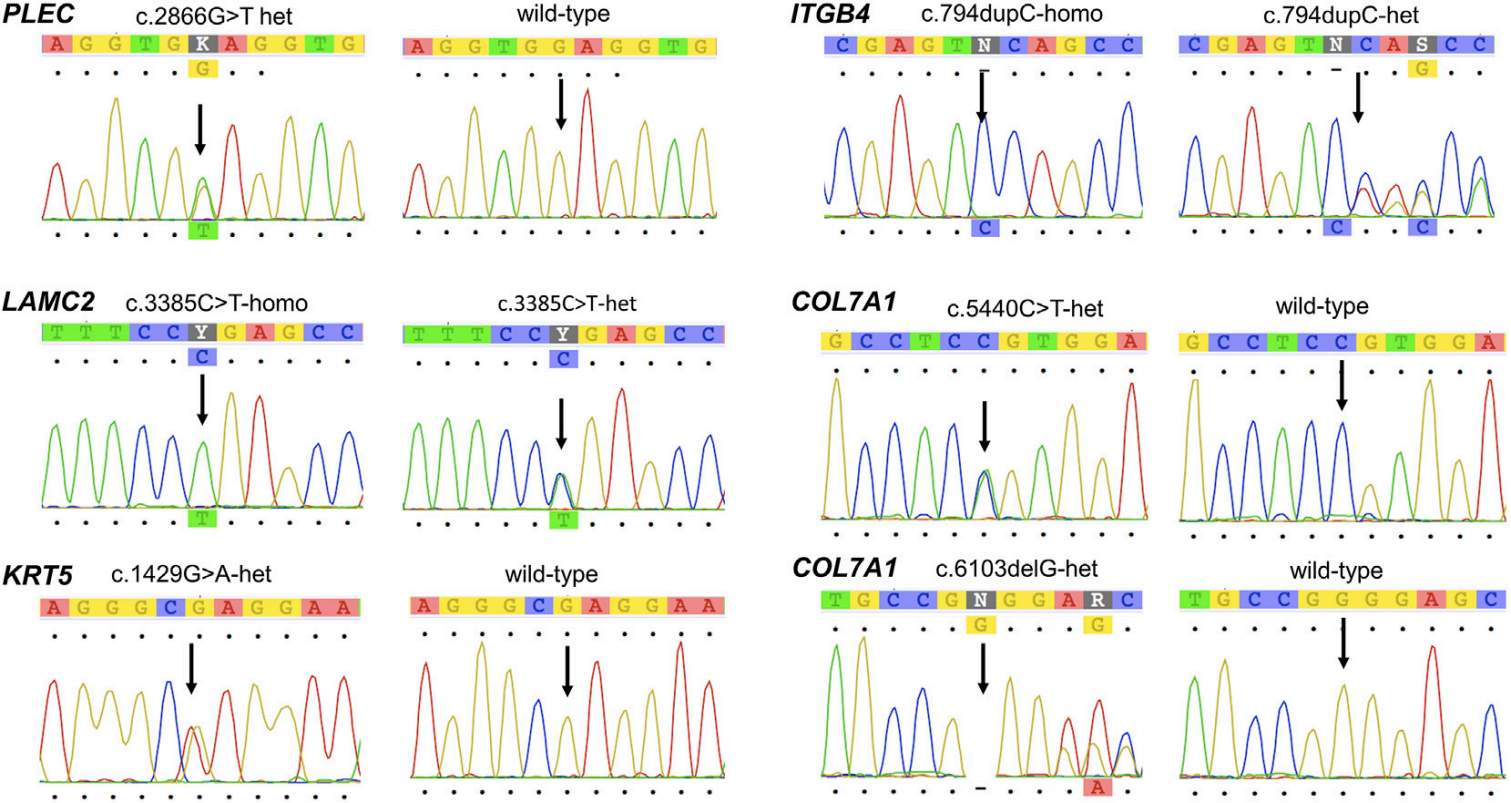
Chromatogram confirming the sequences of the mutations in *PLEC*, *LAMC2*, *KRT5*, *ITGB4*, and *COL7A1* identified in the study.

We identified seven pathogenic/likely pathogenic variants (Figure 3) and 18 unknown significant and benign/likely benign variants (Supplementary Table S3). Details of the disease-responsible candidate variants, including their inheritance status, are shown in Table 2. The novel variants were submitted to ClinVar and assigned reference numbers (Table 2).

**TABLE 2:** Variants and genotypes identified in the present study.

Genetic defects	Patient 1	Patient 2	Patient 3	Patient 4	Patient 5
Gene	*PLEC*	*LAMC2*	*KRT5*	*ITGB4*	*COL7A1*
Variant identified					
Allele 1	c.2536G > T (rs1554713693)	c.3385C > T (rs201307156)	c.1429G > A (rs59190510)	c.794dupC (rs757050033)	c.5440C > T (rs778035441)
Allele 2	Same as allele 1	Same as allele 1	—	Same as allele 1	c.6103delG
ClinVar: Reference	SCV001934588: present study	SCV001983770: present study	VCV000021174: Lalor L, et al., 2018	SCV001934589: present study	VCV001047935.2 (c.5440C > T): Dang N, et al., 2007 and SCV001934590 (c.6103delG): present study
Exon	21	23	7	8	63 (c.5440C > T); 73 (c.6103delG)
Mutant protein	p.Glu846Ter	p.Arg1129Ter	p.Glu477Lys	p.Ala266SerfsTer5	p.Arg1814Cys and p.Glu2035SerfsTer171
Variant classification	Pathogenic (PVS1, PM2, PP3, PP5)	Pathogenic (PVS1, PM2, PP5, PP3)	Pathogenic (PM1, PM2, PP2, PP3, PP5)	Pathogenic (PVS1, PM2, PP3)	Likely pathogenic: c.5440C > T (PM1, PM2, PP2, PP3, PP5) and Pathogenic: c.6103delG (PVS1, PM2, PP3)
Parental study	Both—het c.2536G > T	Both—het c.3385C > T	Both—normal sequence	Both—het c.794dupC	Father - het c.5440C > T; mother - het c.6103delG
Inheritance	AR	AR	AD, *de novo*	AR	AR
gnomAD: MAF	0	0	0	0	0/0
T-REx: MAF	0	0	0	0	0/0
Characteristics of gene					
Total exon	33	23	9	40	119
Amino acid	4,574	1,193	590	1,822	2,944
Ref. gDNA	NC_000,008.10	NC_000,001.10	NC_000,012.11	NC_000,017.10	NC_000,003.12
Ref. mRNA	NM_000,445.5	NM_005,562.3	NM_000,424.4	NM_000,213.5	NM_000,094.4
Ref. protein	NP_000,436.2	NP_005,553.2	NP_000,415.2	NP_000,204.3	NP_000,085.1

AD, autosomal dominant; AR, autosomal recessive; het, heterozygous; MAF, minor allele frequency; Ref., reference sequence; T-REx, Thai reference exome database.

A heterozygous variant of *PLEC*, c.2536G > T, resulting in a change from glutamic acid to stop codon (p.Glu956Ter) was found in the parents of Patient 1 and was not present in his unaffected siblings. Therefore, homozygous *PLEC*:c.2536G > T was inferred as the patient’s genotype, based on segregation analysis using the assumption of autosomal recessive inheritance. This variant has not previously been reported.

A homozygous novel variant in *LAMC2*, c.3385C > T, leading to a stop codon (p.Arg1129Ter) was found in Patient 2. Both parents were heterozygous for the allele, as was a younger unaffected sibling who was born before prenatal testing for familial variants became available.

A heterozygous missense variant of *KRT5*, c.429G > A or p. Glu477Lys, known to cause EB with CAS was detected in Patient 3. As both parents had the normal sequence, this variant was a *de novo* occurrence in the patient.

We detected a novel homozygous single nucleotide insertion, c.794dupC, in *ITGB4* of Patient 4. The parents were heterozygous for the allele.

A genetic compound between a paternally inherited allele, c.5440C > T (p.Arg1814Cys), and a maternally inherited variant, c.6103delG, of *COL7A1* was detected in Patient 5. The c.6103delG was a novel finding.

## Discussion

We identified pathogenic/likely pathogenic variants in five different genes as the causes of EB with CAS in five unrelated Thai families, including four novel recessive alleles in *PLEC*, *LAMC2*, *ITGB4*, and *COL7A1*, and a known dominant mutation in *KRT5*.

A handful of EB cases have been reported in Southeast Asian populations including Thai, Malaysian, Singaporean, and Indonesian (Singalavanija et al., 1994; Wessagowit et al., 2007; Tang et al., 2013; Bishnoi et al., 2021) with only two studies providing molecular data limited to *COL7A1*.(Wessagowit et al., 2007; Bishnoi et al., 2021). One likely reason for the small number of reports on EB with its type/subtype diagnosis in Thailand, at least, is the lack of availability of sophisticated immunohistopathological analyses specific for the disorder.

According to the recent 2020 clinical practice guidelines for laboratory diagnosis of EB in affected neonates, a skin pathologic study is recommended as the first diagnostic step with a genetic analysis performed in parallel (Has et al., 2020b). With the advance of next-generation sequencing (NGS) technology and its rapidly decreasing price, extended genetic testing has become affordable and easier to access for confirmation of disorders with high genetic heterogeneity, such as EB. When applied to our local context, WES was chosen as the first test in the present study.

Several studies using NGS multigene panels for EB diagnosis have reported diagnostic yields of 83–97%, with the variation arising from the characteristics of the examined populations such as ethnicity and presence of consanguinity, as well as the cohort size (40–91 families) and the number (11–49 genes) and details of the genes on the NGS panels (Vahidnezhad et al., 2017; Has et al., 2018; Lucky et al., 2018; Mariath et al., 2019). A study using WES had a 100% detection rate in a cohort of 57 families affected by EB of various types (Yu et al., 2021). The present study yielded 100% detection frequency and revealed genetic heterogeneity in the results, despite the very small cohort. The data support the use of WES as a reasonable alternative method for diagnosis of EB and confirm that it provides very high coverage of target exons/genes. However, the major limitation of genetic testing alone is the lack of histological confirmation of EB, and thus further insights into the dermatopathogenic mechanisms are crucial for deeper research on this disease.

In the present study, four patients, except for the patient with the mildest manifestations, were born prematurely. Furthermore, greater severity of CAS and skin erosion appeared to be associated with greater prematurity. We have noted substantial numbers of premature births among previously reported cases with available data, especially for patients with extensive cutis aplasia and erosion of the skin (Charlesworth et al., 2003; Natsuga et al., 2010b; Chen et al., 2018; Han et al., 2019).

We have described, for the second time, the presence of congenital corneal cloudiness as a primary abnormality of the cornea in two EB patients: Patient 1 with a *PLEC* mutation and Patient 4 with an *ITGB4* mutation. The first case of congenital corneal haziness in EB was recently described in a neonate with RDEB due to *COL7A1* mutations (Sawka et al., 2021). Ocular manifestations in EB usually represent late complications following exposure keratitis and scarring of the eyelids. Our Patient 1 did not show any eye lid abnormality or corneal scarring, while our Patient 4 showed ectropion on one eye but bilateral cloudy cornea, suggesting an underlying mechanism other than physical/rubbing trauma of the cornea.

**FIGURE 4:**
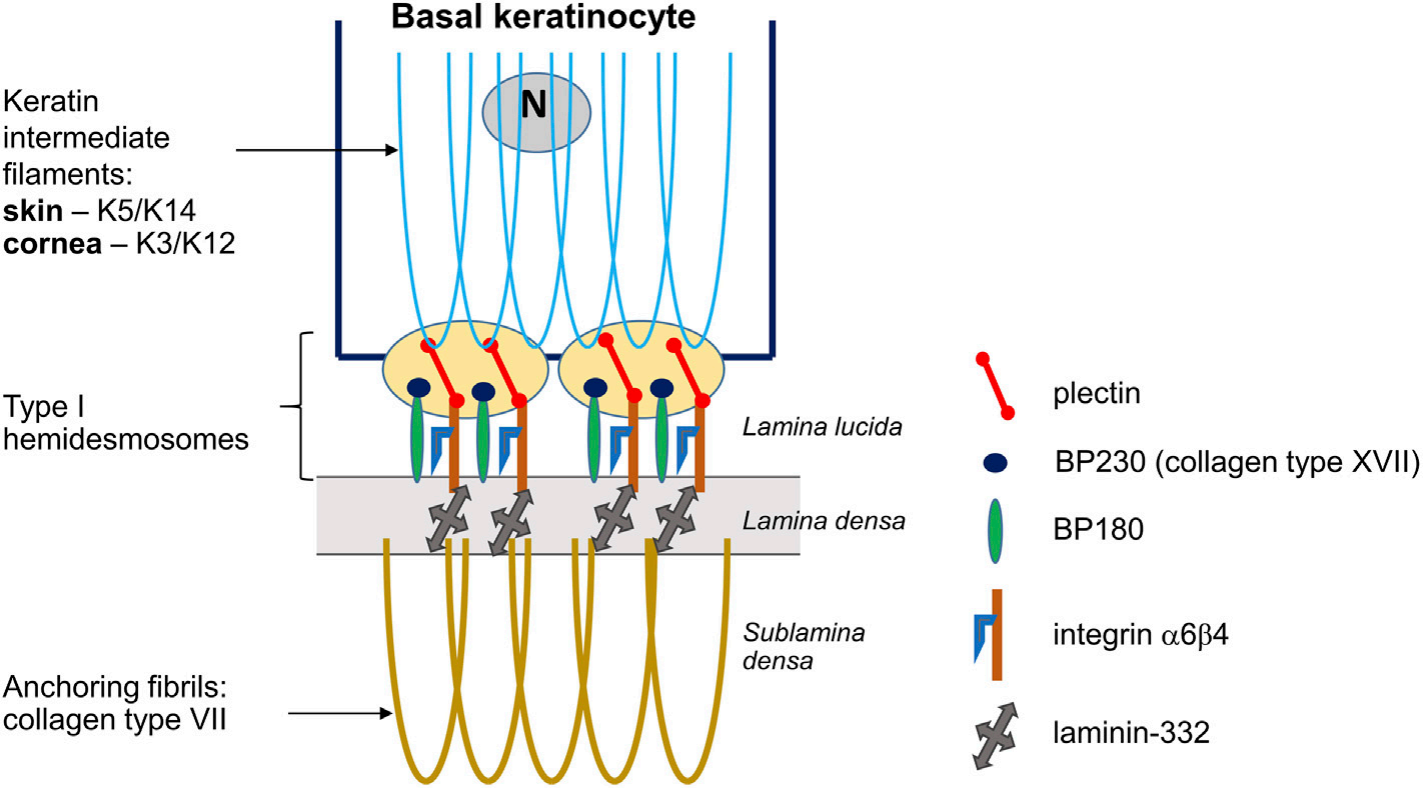
Schematic diagram showing basal keratinocytes and type I hemidesmosomes in the skin and corneal epithelium. Keratin intermediate filaments form within the basal layer. Plectin, a hemidesmosomal protein, directly binds to the intermediate filaments and integrin α6β4, the latter interacts with BP230 and BP180. Laminin-332 binds to integrin α6β4 and the anchoring fibrils composed of collagen type VII.

The skin, oral mucosa, and outer surface of the cornea are stratified squamous epithelium containing type I hemidesmosomes composed of plectin, integrin *α*6*β*4, BP230, and BP180 (collagen type XVII) that link laminin-332 in the basement membrane and lamina densa. The skin-type keratin pair is K5/K14, while the cornea-type keratin pair is K3/K12 (Figure 4) (Walko et al., 2015; McKay et al., 2020). Collagen types VII and IV are also expressed in the epithelial basement membrane (EpBM) of the cornea and skin dermis. Integrin α6β4 was shown to mediate adhesion of basal cells to the corneal EpBM as well as to collagen, laminin, and other extracellular matrix proteins including fibronectins and vitronectin (McKay et al., 2020). Decreased integrin expression led to reduced proliferation of corneal epithelial cells and impaired adhesion to the underlying basement membrane, resulting in ocular surface defects (McKay et al., 2020). A report on the histopathological findings for the anterior cornea in an adult patient with EBS described normal superficial and intermediate corneal epithelia but abnormal structures in the basement membrane, namely fibrocellular pannus deep to the thickened EpBM and multilaminar basement membrane with faulty adhesion complexes (Adamis et al., 1993). Taken together, these data suggest that the congenital corneal cloudiness in EB could arise through primary malformation of the corneal epithelium.

The partial obstruction of the esophagogastic junction in our Patient 4 represents another rare gastrointestinal anomaly in EB, as it has been previously reported in a few cases.(Doi et al., 1986; Cetinkursun et al., 1995; Maman et al., 1998). These gastrointestinal anomalies might have been underdiagnosed because of the early demise of the patients. The PA and esophagogastic atresia could be a result of intraluminal mechanical and/or chemical irritation, leading to mucosal destruction and followed by cicatrization. We further demonstrated the very rare occurrence of a vesicoureteric obstruction with anuria in our Patient 4 with an *ITGB4* mutation (Dang et al., 2008).

*PLEC* encodes plectin protein, which directly interacts with keratin K5/K14 intermediate filaments and also binds to integrin α6β4 and other related proteins to form a multiprotein complex that stabilize the adhesion of basal keratinocytes (epithelial cells) to the underlying basement membrane (Walko et al., 2015). The p. Glu956Ter variant could lead to a severely truncated protein that lack the entire rod domain and the carboxy-terminal globular domain of normal plectin, the latter of which functions as a β4-integrin binding segment and a binding site for keratin intermediate filaments. Therefore, the mutant protein is likely to be nonfunctional or even not produced through activation of nonsense-mediated mRNA decay, resulting in a fatal phenotype (Natsuga et al., 2010a).

Premature truncation (PTC) mutations in *PLEC* were shown to be associated with early lethality in patients with EBS-PA with CAS (Charlesworth et al., 2003; Nakamura et al., 2005; Pfendner and Uitto, 2005; Natsuga, 2015). Three affected siblings described by Charlesworth et al. (Charlesworth et al., 2003) showed similar patterns of extensive skin involvement in scalp/facial regions, PA, hypotonia, and early demise due to a PTC mutation in *PLEC* (c.2727del14 in exon 22 of NM 00445.5. The extreme phenotypes in these siblings were similar to the findings in our Patient 1, who harbored a pathogenic variant in the adjacent exon of *PLEC* (c.2536G > T, last nucleotide in exon 21 (NM_00,445.5). Molecular and histopathologic studies in the siblings demonstrated that the c.2727del14 allele led to PTC and total absence of plectin protein in the skin annexes (Charlesworth et al., 2003). The unusual severity of our Patient 1 and the patients reported by Charlesworth et al. (Charlesworth et al., 2003) could be due to the critical position of the mutations that cause PTC within the plakin globular domain of plectin.

*LAMC2* encodes the laminin γ2 subunit, which forms a heterotrimer with the laminin α3 and laminin β3 subunits to form laminin-332 (Walko et al., 2015). The variant *LAMC2*:c.3385C > T (p.Arg1129Ter) is predicted to lose the carboxy-terminal 64 amino acids, including the distal segment of the coiled-coil domain. *LAMC2* is the only laminin gene found to have mutations in EBS with CAS, with just five pathogenic variants described in previous reports, (Schneider and Muehle, 2008; Hammersen et al., 2016; Mariath et al., 2020), making *LAMC2* the rarest gene underlying the disorder.

Kertin 5 assembles with keratin 14 to form heterodimeric and tetrameric proteins as the principal components of the intermediate filaments in basal keratinocytes that are crucial for mechanical stability (Walko et al., 2015; Khani et al., 2018). *KRT5*:c.429G > A (p.Glu477Lys) was described as the most common mutation in patients affected by EBS-severe with CAS, and was a *de novo* event in the majority of cases (Lalor et al., 2019; Mariath et al., 2020). The mutation is located at the last amino acid in the highly conserved end of the α-helical termination peptides critical for intermediate filament formation. *In silico* modelling showed that the variant led to a conformational change and an altered charge on the surface of the keratin K5/K14 heterodimers, disrupting the stability of the dimerization and thus intermediate filament formation (Lalor et al., 2019). The variant was associated with a high mortality rate (Sathishkumar et al., 2016; Lalor et al., 2019).

The integrin β4 subunit (encoded by *ITGB4*) and integrin α6 subunit form a heterodimeric protein at the lamina lucida of the skin and at the corneal epithelium. *ITGB4*:c.794dupC is expected to result in substitution of alanine with serine at codon 266, followed by a short frameshift before premature termination five residues later, designated p. Ala266SerfsTer5. Thus, the mutant protein lacks more than 80% of the carboxy-terminus, and is likely nonfunctional or not synthesized due to mRNA decay. Mutations of *ITGB4* have been detected throughout the gene. A patient described by Dang et al. (Dang et al., 2008) with c.3903dupC/p.Gly 273Asp (in exon 31/exon 8), exhibited widespread CAS and skin erosion over the scalp/facial/ear regions, severe vesicoureteric obstruction, and anuria, reminiscent of our Patient 4 who also had a mutation of exon 8 encoding the N-terminal extracellular domain of the integrin β4 subunit. These findings indicate possible correlations between mutations in exon 8 and extremely severe phenotypes.

*COL7A1* encodes the α1 subunit of type VII collagen, a major collagenous component of the lamina densa and upper dermis. Type VII collagen forms anchoring fibrils between stratified squamous epithelia and stroma (Chung and Uitto, 2010). Many *COL7A1* variants have been described to cause DDEB and RDEB, depending on the nature and position of the mutations. Specifically, PTC mutations in both *COL7A1* alleles tend to cause the RDEB-severe subtype, while a missense mutation in at least one allele tends to lead to phenotypic variability: intermediate or severe subtypes. In the present study, the genetic compound between a missense allele and a PTC mutation, p. Arg1814Cys/p.Glu2035SerfsTer171, led to a nonlethal form of RDEB-intermediate (self-improving). Most *COL7A1* variants related to EB with CAS are aggregated in the triple-helix domain (Mariath et al., 2020) encoded by exons 31–109 (Christiano et al., 1994). The p. Arg1814Cys was predicted to produce an additional disulfide bond and disturb the secretion of collagen type VII and the stability of the triple helix (Dang et al., 2007).

We did not validate the predicted deleterious effect of the relevant variants by gene expression at neither RNA nor protein level, representing limitation of the present study.

CAS is present in 23% of EB patients (Mariath et al., 2019). Although the exact pathogenesis of CAS in EB remains to be resolved (Chiaverini et al., 2014), an acceptable long-held hypothesis is that CAS results from *in utero* friction/trauma to the limbs (Kanzler et al., 1992). However, the occurrence of similar pattern and severity of CAS within the same family cannot be explained by this assumption. Our data support an alternative hypothesis, recently proposed by several authors, (Charlesworth et al., 2003; Dang et al., 2008; Chiaverini et al., 2014; Lalor et al., 2019; Mariath et al., 2020), that the mutation type and position are the major contributing factors leading to CAS in all EB type/subtype, as evidenced by specific mutations/regions of the genes found to be linked with recurrent patterns of manifestations in different families, and the same severity and patterns of phenotypes seen in affected individuals within the same families.

Consanguinity as a founder effect is not uncommon in upcountry areas of Thailand, as demonstrated by the presence of homozygous variant in three families (Patients 1, 2 and 4) in the present study. The identification of specific mutations allows not only subtype classification and prognostication, but also precise genetic counselling and reproductive choices.

In conclusion, the present study has revealed exceptionally rare phenotypes in EB with CAS, namely congenital corneal cloudiness, esophageal obstruction, and anuria, and extended the genotypic spectrum of EB-related genes. Our data from an underrepresented population in Southeast Asia can further broaden the knowledge and research on EB.

## Data Availability

The datasets presented in this study can be found in online repositories. The names of the repository/repositories and accession number(s) can be found in the article/Supplementary Material.
