# Supplementary material for: Epidermolysis Bullosa With Congenital Absence of Skin: Congenital Corneal Cloudiness and Esophagogastric Obstruction Including Extended Genotypic Spectrum of PLEC, LAMC2, ITGB4 and COL7A1
Source: Front Genet. 2022 Apr 1;13:847150. doi: 10.3389/fgene.2022.847150 (PMC9010945; doi:10.3389/fgene.2022.847150)
Supplement: Supplementary file 1 [file DataSheet1.docx]

Supplementary Material

**Epidermolysis bullosa with congenital absence of skin: congenital corneal cloudiness and esophagogastric obstruction including extended genotypic spectrum of *PLEC*, *LAMC2*, *ITGB4* and *COL7A1***

**Pharuhad Pongmee et al.**

**Supplementary Methods**

Analysis of the raw genome sequencing data, including quality checking (using FastQC package), read alignment (using BWA) to the GRCh37/hg19 human reference genome, variant calling (using SAMTOOLS) and annotation (using ANNOVAR), was conducted in accordance with the Broad Institute best practice guidelines for GATK v3.4 and previous established methods (Van der Auwera et al., 2013), using an open-source bioinformatics tools, BaseSpace Variant Interpreter program (https:variantinterpreter.informatics.illumina.com).

**Reference**

Van der Auwera, G. A., Carneiro, M. O., Hartl, C., Poplin, R., Del Angel, G., Levy-Moonshine, A. et al. (2013). From FastQ data to high confidence variant calls: the Genome Analysis Toolkit best practices pipeline. *Curr Protoc Bioinformatics.* 43, 11 10 11-11 10 33. doi: 10.1002/0471250953.bi1110s43.

**Supplementary Figures and Tables**

**TABLE S1** Primer sequences and PCR conditions

| Gene | Exon | Sequence (5′-3′) | Annealing temperature (°c) | Product size (bp) |
| --- | --- | --- | --- | --- |
| *PLEC* | E21-22F^b^ | CCTGTCCCAGCTATACCTCCT | 59 | 613 |
|  | E21-22R^b^ | AGGGCTACAGTCAGCGTC |  |  |
| *LAMC2* | E23F | GGGTATAGAAGGGCACGGG | 60 | 296 |
|  | E23R | GGGGCAGGTTGTCCCTAATG |  |  |
| *KRT5* | E7F | CTGCCACCCACGATGTACTG | 59 | 638 |
|  | E7R | GGTTGAGCAAAGGACGTGAG |  |  |
| *ITGB4* | E8F | TCCTGTTTCTCAGCGTTGTCA | 59 | 638 |
|  | E8R | CCCACTAGGGCCCACATTT |  |  |
| *COL7A1* | E63F | GGTCTGTGCCTGAGGGATCT | 60 | 402 |
|  | E63R | TGTAGGTGTGCTGGCGTTT |  |  |
|  | E73F | GGAGATCGTGGAGACCTGGG | 60 | 495 |
|  | E73R | TCCCTGAAATGCAAATAGCGG |  |  |

**TABLE S2** Lists of genes analyzed and the coverage and missing regions for the target genes

| Gene | Ref seq. | Total amino acids | Total exons | %Coverage | Missing exons |
| --- | --- | --- | --- | --- | --- |
| ***PLEC*** | NM_000445.5 | 4,574 | 33 | 100.0 |  |
| ***LAMC2*** | NM_005562.3 | 1,193 | 23 | 100.0 |  |
| ***KRT5*** | NM_000424.4 | 590 | 9 | 100.0 |  |
| ***ITGB4*** | NM_000213.5 | 1,822 | 40 | 97.5 | E33 |
| ***COL7A1*** | NM_000094.4 | 2,944 | 119 | 100.0 |  |
| *ATP2C1* | NM_001378687.1 | 919 | 28 | 96.4 | E20 |
| *CDSN* | NM_001264.5 | 529 | 2 | 100.0 |  |
| *COL17A1* | NM_000494.4 | 1,497 | 56 | 96.4 | E2, E17 |
| *CSTA* | NM_005213.4 | 98 | 3 | 100.0 |  |
| *DSG1* | NM_001942.4 | 1,049 | 15 | 100.0 |  |
| *DSG2* | NM_001943.5 | 1,118 | 15 | 100.0 |  |
| *DSG4* | NM_177986.5 | 1,040 | 16 | 100.0 |  |
| *DSP* | NM_004415.4 | 2,871 | 24 | 100.0 |  |
| *DST* | NM_001374736.1 | 7,818 | 104 | 99.0 | E17 |
| *EXPH5* | NM_015065.3 | 1,989 | 6 | 100.0 |  |
| *FERMT1* | NM_017671.5 | 677 | 15 | 100.0 |  |
| *GRIP1* | NM_001366722.1 | 1,128 | 25 | 100.0 |  |
| *ITGA3* | NM_002204.4 | 1,051 | 26 | 96.1 | E26 |
| *ITGA6* | NM_001079818.3 | 1,091 | 25 | 100.0 |  |
| *KLHL24* | NM_017644.3 | 600 | 8 | 100.0 |  |
| *KRT1* | NM_006121.4 | 644 | 9 | 100.0 |  |
| *KRT14* | NM_000526.5 | 472 | 8 | 100.0 |  |
| *LAMA3* | NM_198129.4 | 3,333 | 75 | 98.7 | E13 |
| *LAMB3* | NM_000228.3 | 1,172 | 23 | 100.0 |  |
| *PKP1* | NM_001005337.3 | 726 | 14 | 100.0 |  |
| *TGM5* | NM_201631.4 | 720 | 13 | 100.0 |  |
| Total 26 genes | | | 733 exons | 99.05% (726/733) | 7 exons |

**TABLE S3** List of the variants identified in this study and its classifications of pathogenicity

| Gene | RefSeq transcript ID | Inheritance | Nucleotide change | Protein | Pt1 | Pt2 | Pt3 | Pt4 | Pt5 | Allele frequency | | SNPs ID | ACMG^c^ | Classification |
| --- | --- | --- | --- | --- | --- | --- | --- | --- | --- | --- | --- | --- | --- | --- |
|  |  |  |  |  |  |  |  |  |  | gnomAD^a^ | T-REx^b^ |  |  |  |
| *PLEC* | NM_000445.5 | AD/AR | c.8041T>C | p.Ser2622Pro | NA | - |  | - | - | NA | NA | rs7833924 | BA1, BP4, BP6 | B |
|  | NM_000445.5 | AR | c.2536G>T | p.Glu846Ter | **Hom** | - |  | - | - | 0.000006 | NA | rs1554713693 | PVS1, PM2, PP3, PP5 | **P** |
| *LAMC2* | NM_005562.3 | AR | c.3385C>T | p.Arg1129Stop | - | **Hom** |  | - | - | 0.000004 | NA | rs201307156 | PVS1, PM2, PP3, PP5 | **P** |
| *KRT5* | NM_000424.4 | AD/AR | c.1429G>A | p.Glu477Lys | - | - | **Het** | - | - | NA | NA | rs757050033 | PM1, PM2, PP2, PP3, PP5 | **P** |
|  | NM_000424.4 | AD/AR | c.158G>A | p.Gly53Asp | - | - |  | Het | - | 0.000992 | 0.000 | rs556992218 | PM1, PM2, PP2, BP4 | VUS |
| *ITGB4* | NM_000213.5 | AD/AR | c.2531G>A | p.Arg844His | - | Het |  | - | - | NA | 0.011 | rs140819116 | PM2, PP2 | VUS |
|  | NM_000213.5 | AD/AR | c.794dupC | p.Ala266SerfsTer5 | - | - |  | **Hom** | - | 0.000327 | NA | rs757050033 | PVS1, PM2, PP3 | **P** |
| *COL7A1* | NM_000094.4 | AD/AR | c.8007G>C | p.Gln2669His | NA | - |  | - | - | 0.000028 | NA | rs369881673 | PM1, PM2, PP2, BP4 | VUS |
|  | NM_000094.4 | AD/AR | c.6103delG | p.Glu2035SerfsTer171 | - | - |  |  | **Het** | NA | NA | NA | PVS1, PM2, PP3 | **P** |
|  | NM_000094.4 | AD/AR | c.5440C>T | p.Arg1814Cys | - | - |  | - | **Het** | 0.000087 | NA | rs778035441 | PM1, PM2, PP2, PP3, PP5 | **LP** |
|  | NM_000094.4 | AD/AR | c.4162C>T | p.Arg1388Cys | - | Het |  | - | - | 0.000028 | NA | rs565472139 | PM1, PM2, PP2, BP4 | VUS |
|  | NM_000094.4 | AD/AR | c.3937G>A | p.Ala1313Thr | - | - |  | - | Het | 0.001463 | NA | rs778191454 | PM1, PM2, PP2, BP4, BP6 | LB |
| *COL17A1* | NM_000494.4 | AR | c.2182G>A | p.Gly728Ser | Het | - |  | - | - | NA | NA | NA | PM2, PP3, BP1 | VUS |
|  | NM_000494.4 | AR | c.2163A>G | p.Thr721Thr | NA | - |  | - | - | 0.000012 | NA | rs559497323 | PVS1, PM2, BP4 | **LP** |
| *DST* | NM_001374736.1 | AR | c.15952A>G | p.Asn5318Asp | - | - |  | - | - | NA | NA | NA | PM2, BP1 | VUS |
| *EXPH5* | NM_015065.3 | AR | c.5564T>G | p.Leu1855Arg | - | - |  | - | Het | 0.000992 | 0.003 | rs184537668 | PM2, PP3, BP1 | VUS |
|  | NM_015065.3 | AR | c.3610G>T | p.Ala1204Ser | NA | - |  | - | - | 0.000021 | 0.009 | rs577224198 | PM2, BP1, BP4 | LB |
|  | NM_015065.3 | AR | c.983G>A | p.Arg328Gln | NA | - |  | - | - | 0.025395 | 0.038 | rs11212684 | BS1, BS2, BP1, BP4, BP6 | B |
|  | NM_015065.3 | AR | c.400T>A | p.Ser134Thr | - | - |  | Het | - | 0.000406 | NA | rs145231502 | PM2, BP1, BP4 | LB |
| *FERMT1* | NM_017671.5 | AR | c.1762A>G | p.Asn588Asp | - | Het |  | - | - | 0.000143 | 0.013 | rs185314931 | PM2, PP3, BP1 | VUS |
| *KLHL24* | NM_017644.3 | AD | c.590A>C | p.Asp197Ala | - | - |  | Het | - | NA | 0.038 | rs116961268 | BS1, BS2, BP4 | B |
| *KRT14* | NM_000526.5 | AD/AR | c.1237G>A | p.Ala413Thr | NA | - |  | - | - | 0.021391 | NA | rs59780231 | PM1, PP2, PP5, BA1 | B |
| *LAMA3* | NM_198129.4 | AR | c.8500A>C | p.Ser2834Arg | - | Hom |  | - | - | NA | NA | rs1154233 | PM2, BP1, BP4 | LB |
|  | NM_198129.4 | AR | c.8500A>G | p.Ser2834Gly | Het | - |  | - | - | NA | 1.000 | rs1154233 | BA1, BP1, BP4, BP6 | B |
| *PKP1* | NM_001005337.3 | AR | c.1127C>T | p.Ala376Val | - | Het |  | - | - | NA | NA | NA | PM2, BP1, BP4 | LB |

AD, autosomal dominant; AR, autosomal recessive; B, benign; Het, heterozygous, Hom, homozygous; LB, likely benign; LP, likely pathogenic, P, pathogenic; VUS, variant of uncertain significance.  ^a^ genome aggregation database (<http://gnomad.broadinstitute.org/>); ^b^ Thai Reference Exome Database (<https://trex.nbt.or.th/>); ^c^ Richards, S. et al. Genet Med*.* 17, 405*-*424 (2015)

**FIGURE S1** Pedigree of families 1-5

**
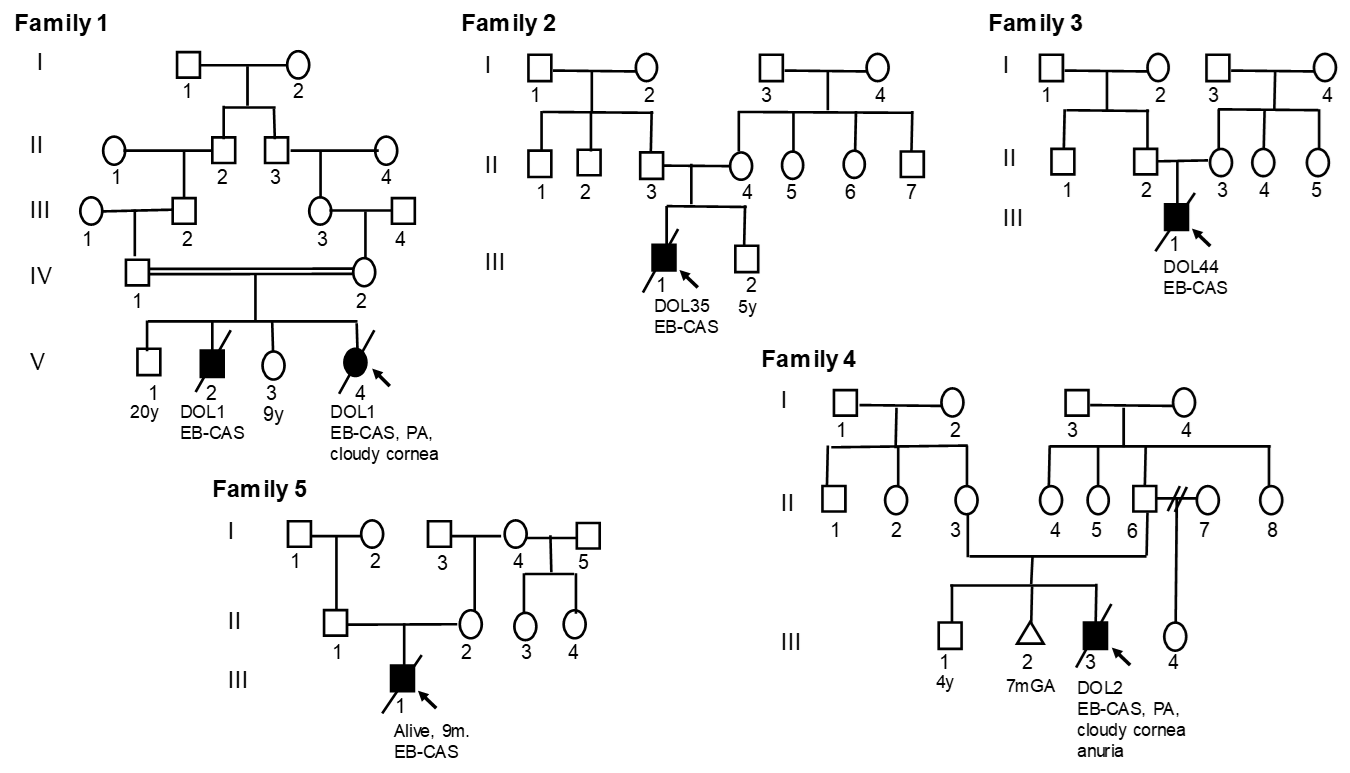
**

**
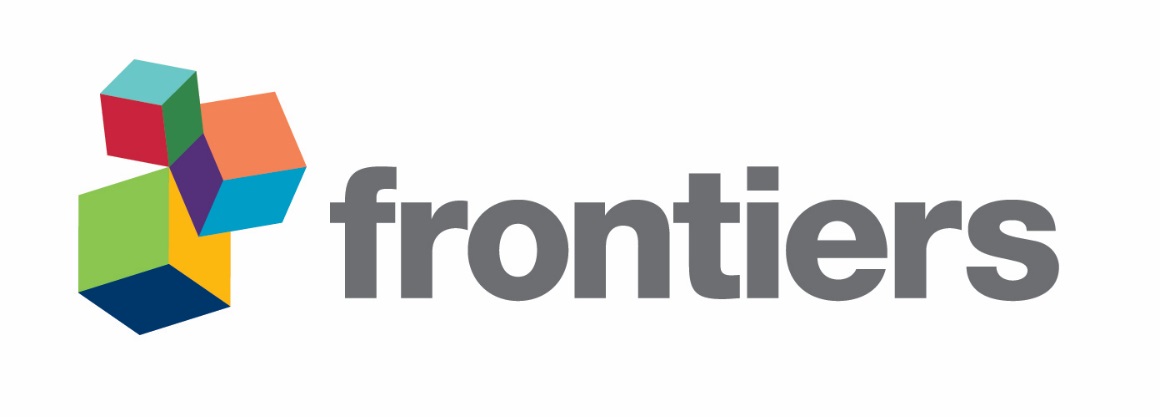
**
